# Effects of Bamboo Expansion on Soil Enzyme Activity and Its Stoichiometric Ratios in Karst Broad-Leaved Forests

**DOI:** 10.3390/biology14121761

**Published:** 2025-12-09

**Authors:** Long Tong, Qingping Zeng, Lijie Chen, Xiaoying Zeng, Ling Shen, Fengling Gan, Minglan Liang, Lixia Chen, Xiaoyan Zhang, Lianghua Qi

**Affiliations:** 1 International Centre for Bamboo and Rattan, Beijing 100102, China; tonglongcq@outlook.com; 2 Chongqing Academy of Forestry, Chongqing 400036, China13983302857@163.com (L.S.); 3 Chongqing Key Laboratory of Forest Ecological Restoration and Utilization in the Three Gorges Reservoir Area, Chongqing 400036, China; 4 Chongqing Key Laboratory of Surface Process and Ecological Restoration in the Three Gorges Reservoir Area/Karst Research Team, Chongqing Key Laboratory of Carbon Cycle and Carbon Regulation of Mountain Ecosystem, School of Geography and Tourism Science, Chongqing Normal University, Chongqing 401331, China; ganfengling@cqnu.edu.cn; 5 Nanchuan District Forestry Industry Development Service Center, Chongqing 408499, China; m18234323932@163.com; 6 Chongqing Jinfo National Nature Reserve Management Affairs Center, Chongqing 408400, China; clx20150115@126.com; 7 Jinfo Mountain Forest Farm, Nanchuan District, Chongqing 408400, China; tangjiajia@126.com

**Keywords:** soil enzyme activity, microbial resource limitation, expansion ratios of bamboo, soil enzymatic stoichiometry, karst regions

## Abstract

The expansion ratio of bamboo forests has significantly influenced soil nutrient cycling in broad-leaved forests through alterations in vegetation composition. However, the extent to which varying expansion ratios of bamboo within broad-leaved forest areas (MRB) affect soil enzyme activities and stoichiometric ratio in karst regions with yellow limestone soil remains poorly understood. Therefore, we examined five MRB levels (<20%, 20–40%, 40–60%, 60–80%, and >80%) and three soil depths (0–20 cm, 20–40 cm, and 40–60 cm) to analyze variations in soil microbial resource limitation within a field experiment conducted in a karst region. The results revealed showed that root morphology was significantly less developed at both low (<20%) and high (≥80%) expansion ratios than at moderate expansion ratios (40–60%) and exhibited a decreasing trend with increasing soil depth. The soil TC (46.43 g·kg^−1^), SOC (80.05 g·kg^−1^) and N:P (0.96) reached their highest values in the 40–60% MRB, whereas the pH (4.61), BD (0.42 g·cm^−3^), AN (20.28 g·kg^−1^) and C:N (45.51) were lowest at 0–20 cm. Moreover, the CBH and the E_C:N_ ratio at 40–60 cm depth in the 0–20% MRB were significantly 2.64 and 1.31 times greater than those at 0–20 cm depth in the 40–60% MRB. Mantel and structural equation modeling (SEM) analyses revealed that soil enzyme activity and stoichiometric ratios are indirectly influenced by soil bulk density (β = −0.156) and root characteristics (β = −0.630). Overall, both C and P limitations are present at the lowest MRB (<20%), whereas other MRB ranges exhibit only P limitation without C limitation. Our results highlight that soil nutrient availability in karst regions of Southwest China is influenced by vegetation structure. These findings provide a scientific foundation for achieving the green and sustainable management of bamboo forests within broad-leaved forest ecosystems.

## Simple Summary

This study investigated the effects of different expansion ratios of bamboo within broad-leaved forest (MRB) and soil depths on soil microbial resource limitation in a field experiment conducted in a karst region. The results demonstrated that soil enzyme activity and stoichiometric ratios are indirectly influenced by soil bulk density and root characteristics. Overall, both carbon (C) and phosphorus (P) limitations were observed at the lowest MRB level (<20%), whereas higher MRB levels exhibited P limitation exclusively, with no significant C limitation. These findings provide a scientific basis for promoting green and sustainable management practices in bamboo-invaded broad-leaved forest ecosystems.

## 1. Introduction

Bamboo is a renewable and versatile forest resource primarily found in tropical and subtropical regions, although some species thrive in temperate and cold climates [1,2]. As one of the leading bamboo producers in the world, China has approximately 6.01 million hectares of bamboo forest and leads globally in terms of bamboo species diversity, growth performance, and product output [3]. In alignment with the 2030 Sustainable Development Goals, China has enhanced environmental conservation efforts by curbing the overexploitation of natural forests and promoting the rapid expansion of bamboo and broad-leaved mixed forest cultivation in major bamboo-producing regions [4,5,6]. Relevant studies have demonstrated that, compared with pure bamboo forests, mixed bamboo forests present greater levels of soil fertility, forest health, and productivity [7,8]. Nevertheless, there is a relative lack of comprehensive management strategies for broad-leaved trees within forest stands, resulting in insufficient scientific and practical guidance for the sustainable management of bamboo and broad-leaved mixed forests. To achieve sustainable forest management, it is essential to consider both ecological and economic benefits when determining the appropriate expansion ratios of bamboo within broad-leaved forests.

Moreover, soil enzyme activity and stoichiometry can clarify the relationship between soil nutrient availability and microbial nutritional demands and reveal the balance between microbial biomass stoichiometry and soil stoichiometry [9,10]. Some studies have demonstrated that, compared with pure forests, mixed-species forests exhibit a greater ability to regulate soil microbial communities and soil enzyme activities through modifications in soil properties [11]. Moreover, several studies have demonstrated that the soil enzymatic activities of urease, sucrase, and catalase progressively decrease with increasing soil depth across various mixed ratios in bamboo and broad-leaved mixed forests [3,12]. The presence of diverse soil fauna and microbial communities stimulates their metabolic activity, leading to markedly higher soil enzyme activity at the soil surface than in deeper layers [13,14]. Therefore, the root-mediated input of organic matter from a single tree species, as influenced by competitive interactions in mixed stands, clearly exerts distinct effects on soil nutrient cycling and microbial-driven soil enzyme production. However, research exploring the impact of varying expansion ratios of bamboo and broad-leaved mixed forests on soil enzyme activity across different soil depths at small-scale spatial levels remains limited.

Additionally, on the basis of the relationship between soil enzyme stoichiometry and soil chemical stoichiometry, microbial resource limitation and its primary influencing factors can be assessed under varying expansion ratios in bamboo and broad-leaved mixed forests [15,16]. Research has demonstrated that the expansion ratio (canopy width ratio) of bamboo in small-scale broad-leaved forests plays a pivotal role in significantly influencing soil nutrients and the microbial community structure [17,18]. Furthermore, the fine root traits of bamboo forests enable the continuous exudation of various inorganic substances into the soil, thereby influencing soil enzyme activity and interactions with soil enzymes during plant growth [19,20]. Consequently, changes in soil enzyme activities are frequently employed as indicators to evaluate improvements in soil environmental conditions and plant growth status. Therefore, investigating root functional traits, soil enzyme activities, and their associated nutrient limitations across different expansion ratios of bamboo forests is highly important for comprehensively understanding the adaptive mechanisms of these traits and their influence on soil ecological processes.

The United Nations Sustainable Development Goals for 2030 aimed at improving the major challenges the world is facing, such as poverty, inequality, climate change and environmental degradation, during the period from 2015–2030 [21,22]. This project emphasizes the imperative to halt land degradation and prevent biodiversity loss. Studies have shown that prolonged and excessive use of agrochemicals by bamboo farmers, coupled with disregard for the ecological and sociocultural values of forests, has led to declining soil fertility, accelerated soil erosion, and progressive degradation of the ecological environment [6,23,24]. Therefore, to promote a more stable and efficient forest ecosystem that balances economic benefits with the Sustainable Development Goals for 2030, it is necessary to explore the specific mechanisms by which bamboo forest expansion influences soil enzyme activities and their associated nutrient limitations in mixed bamboo and broad-leaved forests.

*Chimonobambusa utilis* is a species of bamboo belonging to the genus *Chimonobambusa* Makino within the family Poaceae (Gramineae), which has specific requirements for soil and climate conditions and is typically found growing in regions with altitudes ranging from 800–2500 m [25,26]. Driven by the relentless pursuit of maximum economic gain, they focus exclusively on managing pure *C. utilis* in the broad-leaved forest within these ecosystems [21,27]. Researchers have conducted numerous studies on the soil nutrient limitation dynamics associated with the expansion of *C. utilis* [28,29,30]. However, the relationships between root elements and related microbial resource limitations during the expansion of *C. utilis* in broad-leaved forests remain unclear, which limits the understanding of the mechanism of the effects of bamboo expansion on soil enzyme activities and their stoichiometric ratios in karst regions. Therefore, we selected the expansion ratios of bamboo within broad-leaved mixed forests (MRB) as well as soil depth as key environmental variables to assess the dynamic patterns and driving mechanisms of soil enzyme activities and their stoichiometry on the Jinfo karst Mountain.

We formulated the following hypotheses: (1) Increased expansion ratios of bamboo and soil depth reduce soil enzyme activities, thereby exacerbating microbial C and P limitations; (2) the dominance of bamboo expansion, particularly under relatively high MRB conditions, is likely to reduce soil enzyme activities and exacerbate microbial resource limitations. Therefore, we focused on evaluating (ⅰ) the effects of MRB and soil depth changes on root morphological characteristics, soil nutrients, enzyme activities, and stoichiometry and (ⅱ) the relationships between soil enzymes and soil nutrients in relation to microbial resource limitations.

## 2. Materials and Methods

### 2.1. Site Description

The study area is located within the Jinfo Mountain Karst World Heritage Site (106°54′–107°27′ E, 28°46′–29°38′ N), China [31]. The region features distinctive limestone karst formations, with mountains encompassing 98.79% of the land and boasting a maximum elevation drop of 1600 m. The highest peak reaches an elevation of 2238 m, whereas the lowest point lies at an elevation of 650 m above sea level [32]. The Jinfo Mountain Nature Reserve represents a typical subtropical humid monsoon climate zone characterized by distinct vertical climatic variations and four well-defined seasons. The reserve receives an average annual rainfall of approximately 1431 mm, with an average annual temperature of 8.3 °C and approximately 1173 h of sunshine per year. It is distinguished by limited sunlight exposure, frequent cloud cover, high humidity levels, and abundant precipitation [33]. Owing to the interaction of biological, climatic, and geological factors, the vertical distribution zone spectrum of soil exhibits distinct zonal and regional patterns. The region harbors a diverse vegetation community and boasts abundant natural resources, providing optimal conditions for the proliferation of numerous rare animal species. Its primary focus lies in safeguarding the subtropical forest ecosystem, with silver fir, forest musk deer, and black langurs serving as key conservation targets [5]. Additionally, *C. utilis* constitute a significant component of the resources in the Jinfo Mountain area, with its distribution ranging from 1300–2238 m above sea level. The *C. utilis* bamboo forest covers an extensive area of over 14,000 hectares, of which Nanchuan accounts for 10,033.05 hectares, representing 60% of the total forested area in Jinfo Mountain. Moreover, locally produced bamboo shoots serve as a vital economic resource for residents [34].

### 2.2. Experimental Design

**Figure 1:**
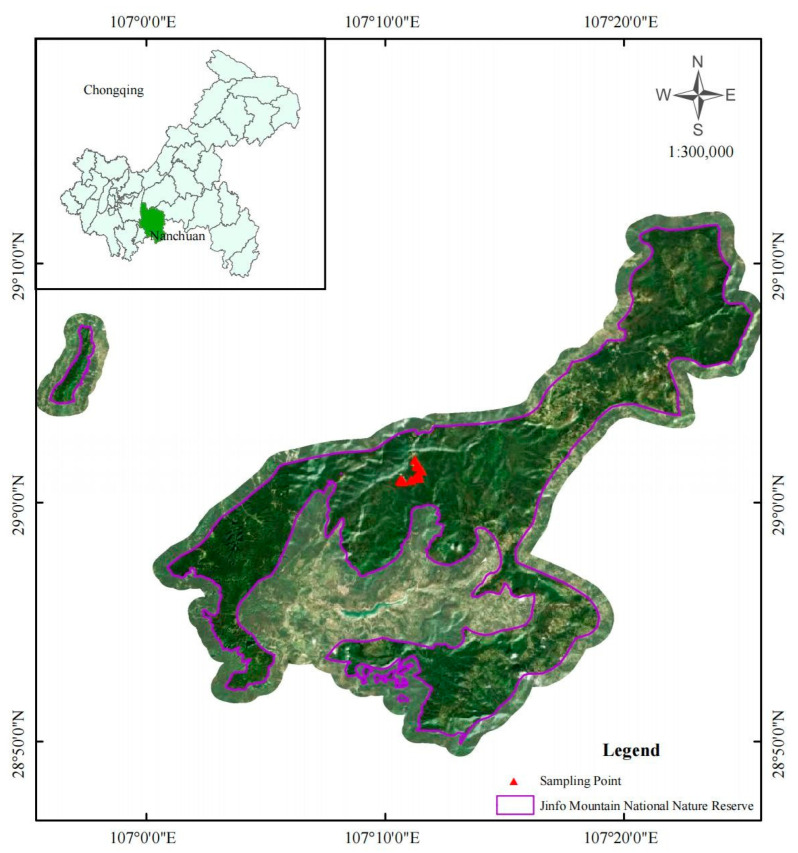
Sampling sites along with expansion ratios of bamboo within the broad-leaved forest.

From August to September 2024, consistent site conditions were used to select mixed bamboo and broad-leaved forests with comparable management levels, stand site qualities, elevations, and other relevant factors. A comprehensive survey of the protected area served as funding for this selection. To establish representative plots within their respective distribution areas, a systematic plot establishment approach was employed to set up five types of mixed bamboo and broad-leaved forest plots, each measuring 15 m × 15 m (Figure 1). According to the survey, the selected sample plots of *C. utilis* were uniformly 2 to 4 years old, representing 60% of all *C. utilis*. In certain broad-leaved forest stands co-occurring with *C. utilis* and other associated species, young and middle-aged broad-leaved trees are also present. The stand ages of these forests align with the growth cycles of their associated understory vegetation, reflecting a multicohort structure characterized by the coexistence of multiple age classes. The expansion ratio of bamboo was determined on the basis of the ratio of the chest height area between the bamboo and broad-leaved trees, specifically dividing the expansion ratios of bamboo within the broad-leaved forest according to their relative significance in terms of the chest height area. Specifically, the expansion ratios of bamboo within the broad-leaved forest (MRB) were classified as <20%, 20–40%, 40–60%, 60–80%, and >80%, which correspond to the cover ratios of *C. utilis* in stands with similar compositions of broad-leaved tree species, including *Quercus myrsinifolia*, *Quercus engleriana*, *Castanopsis platyacantha*, *Rhododendron coeloneurum*, and *Machilus nanmu* [18]. Moreover, the bamboo expansion ratio was measured in five replicate plots, with five soil samples collected per plot according to the “S” soil sampling method. This corresponds to 5 bamboo expansion ratios × 5 replicate plots per expansion ratio × 5 replicate soil samples per plot × 3 soil depths per soil sample, resulting in a total of 375 soil samples collected in this study. To ensure precise replication and avoid potential interference, the minimum distance between adjacent plots was set to 50 m. The sample site is situated on a south- or southeast-facing slope at the midslope level. The slope angle ranges from 30° to 43°, whereas the elevation varies between 2100 m and 2238 m. Being located within a national nature reserve, it experiences minimal human disturbance. During the establishment of each sample plot, the starting point was designated the southwest corner. A compass is utilized for directional alignment, while distances are measured via a tape measure. PVC pipes were employed to mark the four corners of each plot, with their respective serial numbers inscribed on them. Additionally, warning ropes are attached to connect all four corners of every sample plot [35].

### 2.3. Sample Collections

In the dynamic mixed bamboo and broad-leaved forest, where a diverse assemblage of lush broad-leaved trees intertwines with towering square bamboo, soil samples were systematically collected from eight strategically chosen points via the *S*-shaped sampling technique. Five two-year-old standard bamboo culms were selected from the sample plot on the basis of average diameter at breast height and total height, as this age class has the most vigorous physiological activity and best represents the overall growth status of the stand. Three sampling points were established along a 20 cm radius arc centered on the reference bamboo plant. Soil samples were collected via a root auger at three depth intervals: 0–20 cm, 20–40 cm, and 40–60 cm [12]. Following the removal of the soil core from the roots, the root system was gently agitated to remove loosely adherent soil particles. All the root systems originating from the same soil layer were immediately pooled, and the resulting composite samples were sealed in bags and stored in a portable refrigerator at 4 °C until further analysis.

From each plot, the soil samples obtained at the eight sampling points were carefully homogenized layer by layer to produce a representative composite sample for the entire plot. The collected soil samples were subsequently systematically divided into three precisely equal portions via the quartering method to ensure uniformity and reliability. After the surface litter and gravel were carefully removed, each composite sample was separated into two distinct fractions via a 2 mm mesh sieve. One fraction, consisting of fresh soil, was stored at −20 °C in a refrigerator for subsequent determination of soil enzyme activities. The other fraction was air-dried under controlled conditions and preserved for analysis of the soil physical and chemical properties [36].

The cleaned root systems were scanned in grayscale using an EPSON Perfection V700 scanner (Seiko Epson Corporation, Suwa City, Nagano Prefecture, Japan). The morphological parameters, including the root diameter, root length, root surface area, root volume, and number of root tips, were then analyzed from the scanned images. The surface moisture was removed by air-drying after scanning, and the samples were then weighed to determine the fresh root weight (FRW).

### 2.4. Laboratory Analysis

The root systems were subsequently sieved, and the roots designated for individual tensile testing were separated. The remaining root material was oven-dried at 65 °C until a constant mass was achieved. The dry root weight (DRW) was recorded, and the fine root biomass density (RBD) was calculated. Following these procedures, the fresh weight, dry weight, root count, and morphological characteristics were measured.

The root length density (RLD), root surface area density (RSAD), and root volume density (RVD) were then calculated on the basis of the root length, surface area, and volume, respectively. The calculation method is as follows:

RBD = DRW × 10^6^/[π × (d/2)^2^ × h] (1)

RLD = RL/V(2)

RSAD = RSA/V(3)

RVR = RV/V(4)

where RBD denotes the fine root biomass density (g·m^−3^), defined in this study as the dry mass of all living fine roots per unit volume; DRW represents the dry root weight (g); d refers to the diameter of the root coring drill (7 cm); h indicates the height of the root core (10 cm); RL denotes the root length (cm); V represents the volume of the root core sample (dm^3^); RSA refers to the root surface area (cm^2^); and RV denotes the root volume (cm^3^).

The soil pH was measured via a high-precision pH meter (FE28-Standard, METTLER TOLEDO, Greifensee, Switzerland), and the soil bulk density (BD) was determined via the ring knife method [14]. Soil organic carbon (SOC) was quantified via potassium dichromate oxidation with external heating. The soil total carbon (TC) content was analyzed via an elemental analyzer (Elementar Analysensysteme GmbH, Langenselbold, Germany) [11]. The soil total nitrogen (TN) content was determined via a fully automated discrete chemical analyzer (Alliance Smart-Chem 200, Frepillon, France), and the available nitrogen (AN) content was assessed via calcium chloride extraction combined with ultraviolet spectrophotometry [6]. Soil total phosphorus (TP) was measured through sulfuric acid-perchloric acid digestion followed by molybdenum antimony anticolorimetric analysis, and the available phosphorus (AP) was determined via sodium bicarbonate extraction followed by molybdenum antimony anticolorimetric analysis [2].

The soil enzyme activities were measured via an improved microplate fluorescence method [3]. Soil samples collected from different field depths were carefully homogenized through a standardized protocol to minimize within-depth variability and enhance representativeness by reducing the influence of localized variations in moisture or bulk density. Furthermore, measurement consistency and reliability for multiple hydrolase activities were ensured through adherence to the standardization procedures specified in ISO/TS 22939:2019 [37], including control of the incubation time, substrate concentration, and other critical parameters. Specifically, the carbon-acquiring enzymes include β-glucosaminidase (BG) and cellobiosidase (CBH); the nitrogen-acquiring enzymes include β-1,4-N-acetylglucosaminidase (NAG) and L-leucine aminopeptidase (LAP); and the phosphorus-acquiring enzyme is alkaline phosphatase (AKP) [13]. Among these enzymes, the labeled substrate for BG is 4-methylumbelliferyl-β-D-glucoside; for CBH, it is 4-methylumbelliferyl-β-D-cellobioside; for NAG, it is 4-methylumbelliferyl-N-acetyl-β-D-glucosaminide; for LAP, it is L-leucine-7-amido-4-methylcoumarin hydrochloride (7-AMC); and for AKP, it is 4-methylumbelliferyl phosphate [38]. The concise procedure for determination is as follows: Accurately weigh 1 g of fresh soil into a 200 mL sterilized container (e.g., Lock & Lock brand: Seoul, Republic of Korea). Subsequently, 125 mL of 50 mmol·L^−1^ sodium acetate buffer (pH = 5.0) was added [39]. The mixture was homogenized thoroughly by magnetic stirring for 5 min. A precision pipette was used to dispense 250 μL of buffer, 200 μL of soil homogenate sample, 50 μL of standard substance, and 50 μL of substrate into the designated wells of a 96-well microplate. The microplate was incubated in complete darkness at a controlled temperature of 25 °C for 4 h. After incubation, 10 μL of 1 mol·L^−1^ sodium hydroxide solution was added to terminate the reaction. The fluorescence intensity was then measured via a high-performance multifunctional microplate reader (Synergy H4, BioTek, Winooski, VT, USA). Each soil sample was analyzed in six replicates to ensure data reliability. Soil enzyme activity is expressed as the amount of substrate decomposed per unit dry soil weight and per unit time, quantified in nanomoles per hour per gram (nmol·h^−1^·g^−1^).

### 2.5. Calculation of Microbial Resource Limitation

The variations in the soil enzyme activities of (LAP + NAG) or NAG serve as robust indicators of nitrogen resource demands by soil microorganisms, and the ratios of (CBH + BG): (NAG + LAP): AP or BG: NAG: AP effectively represent the ecological stoichiometry of soil extracellular enzymes [40]. Therefore, in this study, the soil enzyme activities of LAP and NAG were precisely quantified, and their combined value of (LAP + NAG) was utilized for subsequent computation and analysis. The following formula was used to evaluate the soil enzyme stoichiometry:

E_C:N_ = (CBH + BG)/(NAG + LAP) (5)

E_C:P_ = (CBH + BG)/AKP (6)

E_N:P_ = (NAG + LAP)/AKP (7)

where CBH stands for cellobiosidase; BG stands for β-glucosidase; NAG represents nitrogen-acquiring enzymes comprising β-1,4-N-acetylglucosaminidase; LAP stands for L-leucine aminopeptidase; and AKP represents alkaline phosphatase.

Some studies have demonstrated that the evaluation of microbial resource limitations is performed through the analysis of enzyme activity ratios [4]. Specifically, a relatively high (CBH + BG)/(NAG + LAP) ratio indicates N limitation, whereas the (NAG + LAP)/AKP ratio reflects N and P limitations.

In addition, the soil extracellular enzyme C, N, and P stoichiometric ratios can be analyzed through vector analysis, which allows for a more precise quantification of the limitations on soil microbial C, N, and P metabolism. The ratio of C relative to nutrient acquisition can be quantified by the vector length, whereas the relative P to N acquisition can be expressed through the vector angle. A longer vector length results in a significantly greater limitation for soil microbial C. When the vector angle is less than 45°, a decreasing value enhances the soil microbial C limitation; conversely, when the vector angle exceeds 45°, an increasing value maximizes the soil microbial P limitation to its fullest extent [41]. The calculation formula for assessing soil microbial nutrient limitations is as follows:

(8) $$\mathrm{Vector}\;\mathrm{length}\;=\;\sqrt{{\left(\frac{\mathrm{In}\left(\mathrm{BG}+\mathrm{CBH}\right)}{\mathrm{In}\left(\mathrm{NAG}+\mathrm{LAP}\right)}\right)}^{2}\;+\;{\left(\frac{\mathrm{In}\left(\mathrm{BG}+\mathrm{CBH}\right)}{\mathrm{In}\;\mathrm{AKP}}\right)}^{2}}$$

Vector angle = Degrees (ATAN2(In (BG + CBH)/In AKP), (In (BG + CBH)/In (NAG + LAP)))(9)

where the vector length denotes the magnitude of the vector and the vector angle indicates the direction of the vector.

### 2.6. Statistical Analysis

The data are presented as the means ± standard deviations (SDs). The influence of soil depth and the expansion ratio of bamboo and broad-leaved forests on variations in soil enzyme activity was assessed via analysis of variance (ANOVA). Significant differences were identified through least significant difference (LSD) tests at *p* < 0.05. Additionally, Pearson correlation analysis was employed to elucidate the intricate relationships among soil nutrient characteristics, enzyme activity, and stoichiometric ratios. All the statistical analyses were conducted via SPSS 25.0, while Origin 24.0 was utilized for the creation of relevant graphical representations [9].

## 3. Results

### 3.1. Root Morphological Characteristics

An analysis of fine root systems in bamboo forests across different soil depths in high-altitude karst regions revealed that the FRW, RLD, RSAD, and RVD decreased with increasing soil depth (Table 1). This finding indicates that the root system is primarily concentrated in the topsoil layer, particularly within the depth range of 0–20 cm. In contrast, the RBD gradually increased with increasing soil depth, indicating that root-associated microbial biomass is predominantly concentrated in deeper soil layers. The DRW sharply decreased at a soil depth of 40–60 cm, decreasing by 88.21% and 88.32%, respectively, compared with the surface soil layers at 0–20 cm and 20–40 cm. In addition, the FRW, DRW, RLD, RSAD, and RVD reached their minimum values when the bamboo expansion ratio was less than 20%. In contrast, FRW, RLD, and RSAD achieved their peak values when the expansion ratio ranged between 20% and 40%. Furthermore, FRW, RLD, and RSAD initially gradually decreased, followed by a sharp decrease when the bamboo expansion ratio exceeded 80%. The RBDs in the 40–60% MBR were 75.78%, 7.84%, 29.95%, and 70.49% greater than those in the <20%, 20–40%, 60–80%, and >80% MRBs, respectively. Moreover, all the root characteristics reached their significantly lowest values at the 40–60 cm soil depth when the expansion ratio of the bamboo exceeded 80%, indicating that the root system of highly expanded bamboo forests gradually decreased with increasing soil depth.

### 3.2. Soil Nutrient Variables and Their Stoichiometry

**Figure 2:**
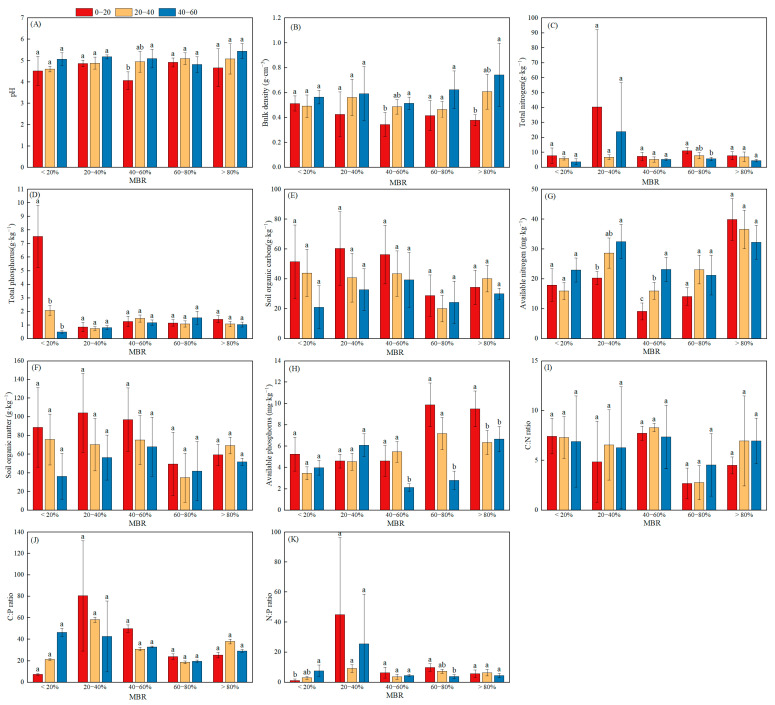
Soil nutrient variables and their stoichiometry with the expansion ratio of bamboo within the broad-leaved forest. (**A**) pH; (**B**) Bulk density; (**C**) Total nitrogen; (**D**) Total phosphorus; (**E**) Soil organic carbon; (**F**) Soil organic matter; (**G**) Available nitrogen; (**H**) Available phosphorus; (**I**) C:N ratio; (**J**) C:P ratio; (**K**) N:P ratio. Different lowercase letters indicate significant differences between different patches of the same soil depth (*p* < 0.05).

The results revealed that the expansion ratios of bamboo within the broad-leaved forest (MRB) and soil depth significantly influenced the soil nutrient variables (Figure 2). PH and BD were significantly greater at the 40–60 cm soil depth in the >80% MBR, whereas SOC, OM, TN, C:P, and N:P were significantly greater at the 0–20 cm soil depth in the 20–40% MBR. In addition, across different soil depths, the average C:P and N:P ratios reached their highest values in the 20–40% MRB, whereas the average SOC and OM contents reached their peak levels in the 40–60% MRB. Across the entire range of the MRB, the soil SOC and OM contents ranged from 20.16 to 60.42 g·kg^−1^ and from 34.76 to 104.17 g·kg^−1^, respectively, which significantly increased from <20% MRB to 40–60% MRB, sharply decreased from 60–80% MRB, and subsequently increased from 60–80% MRB to >80% MRB. Furthermore, compared with those at the other soil depths and MRB levels, the soil TN content significantly increased, whereas the C:P and N:P ratios reached their minimum values at the 0–20 cm soil depth under conditions of <20% MRB. Conversely, the trend for the soil TP content contrasted with those of the soil SOC and OM contents, which significantly decreased from <20% MRB to 20–40% MRB, followed by a decrease from 40–60% to >80% MRB. In general, the soil TC (46.43 g·kg^−1^), SOC (80.05 g·kg^−1^) and N:P (0.96) contents reached their maximum values within the 40–60% bamboo expansion ratio, indicating increased biochemical storage under moderate expansion conditions. Moreover, pH (4.61), BD (0.42 g·cm^−3^), AN (20.28 g·kg^−1^) and C:N (45.51) reached their minimum values in the 0–20 cm soil layer, demonstrating a distinct stratification of soil properties across both the expansion ratio and depth.

### 3.3. Soil Enzyme Activities and Their Stoichiometry

**Figure 3:**
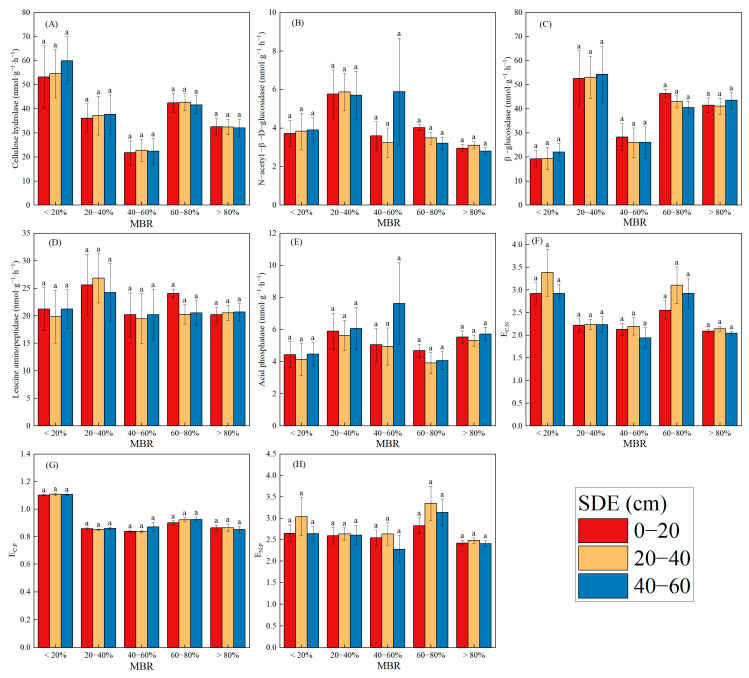
Soil enzyme activities and their stoichiometry at different soil depths and expansion ratios of bamboo within the broad-leaved forest. (**A**) Cellulose hydrolase; (**B**) N-acetyl-β-D-glucosidase; (**C**) β-glucosidase; (**D**) Leucine aminopeptidase; (**E**) Acid phosphorus; (**F**) E_C:N_; (**G**) E_C:P_; (**H**) E_N:P_. Different lowercase letters indicate significant differences between different patches of the same soil depth (*p* < 0.05).

The expansion ratios of bamboo within broad-leaved mixed forests (MRB), soil depth, and their interactions significantly influenced the majority of the soil enzymatic activities and their stoichiometric ratios (Figure 3). In addition to CBH, E_C:P_, and E_C:N_ reaching their maximum values when the MRB is less than 20%, most soil enzymatic activities reach their highest levels within the 20–40% MRB range, indicating a distinct pattern of biochemical activity distribution across bamboo expansion. The minimum values for CBH, LAP, and the ratios of E_C:P_, E_C:N_, and E_N:P_ are observed at 40–60% MRB. An MRB range of 20–40% is clearly optimal for enhancing soil enzyme activity; however, exceeding this range results in a decline in soil enzyme activity. Within the lowest MRB (<20%), soil enzymatic activities increased with increasing soil depth, indicating increased microbial activity in deeper layers under minimal expansion conditions. In contrast, for the 60–80% MRB range, enzymatic activities decreased with depth, suggesting that microbial function in the subsoil was suppressed due to intensified bamboo expansion. Additionally, the E_C:P_ and E_N:P_ ratios were highest at a soil depth of 20–40 cm for most of the MRB, whereas the E_C:N_ ratio was highest at 40–60 cm for the majority of the MRB. However, for 40–60% of the MRB, the E_C:P_ and E_N:P_ ratios were highest at the 0–20 cm soil depth. For the 60–80% MRB, the soil enzymatic activities were more pronounced in the surface soil, suggesting enhanced microbial metabolic activity under moderate expansion conditions.

### 3.4. Linkage Between Soil Enzymes and Soil Nutrients

**Figure 4:**
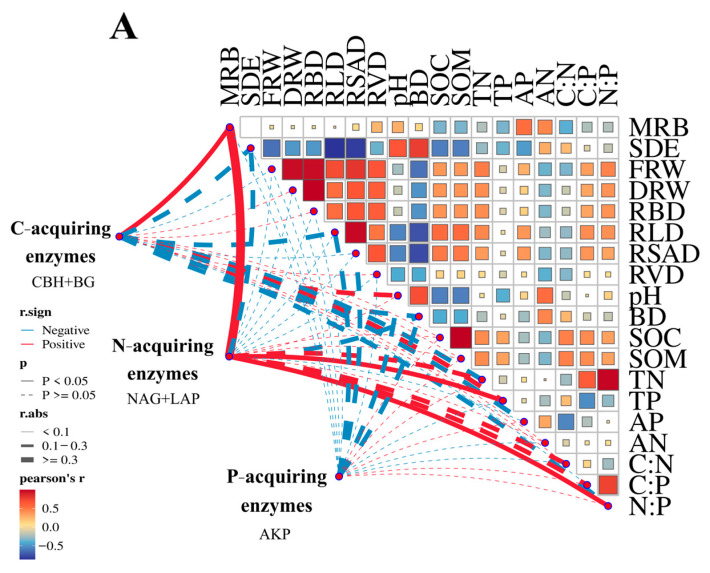

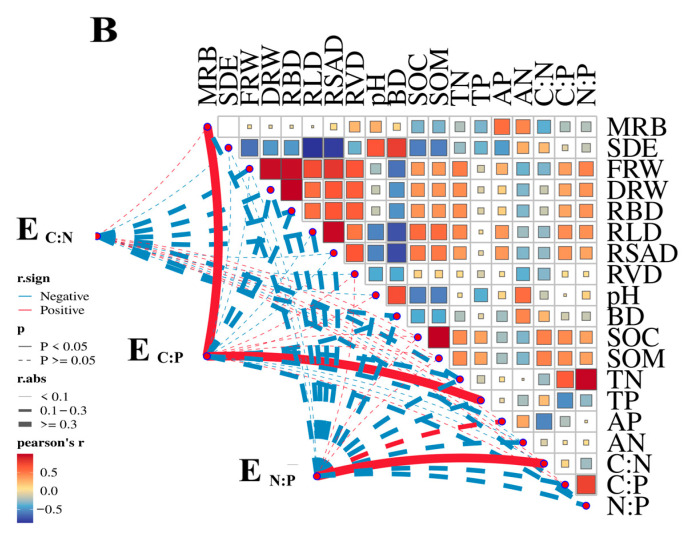
Diagrams demonstrating significant positive and negative correlations between soil variables and soil enzyme activities, as well as the soil enzyme stoichiometric ratio (**A**,**B**).

**Figure 5:**
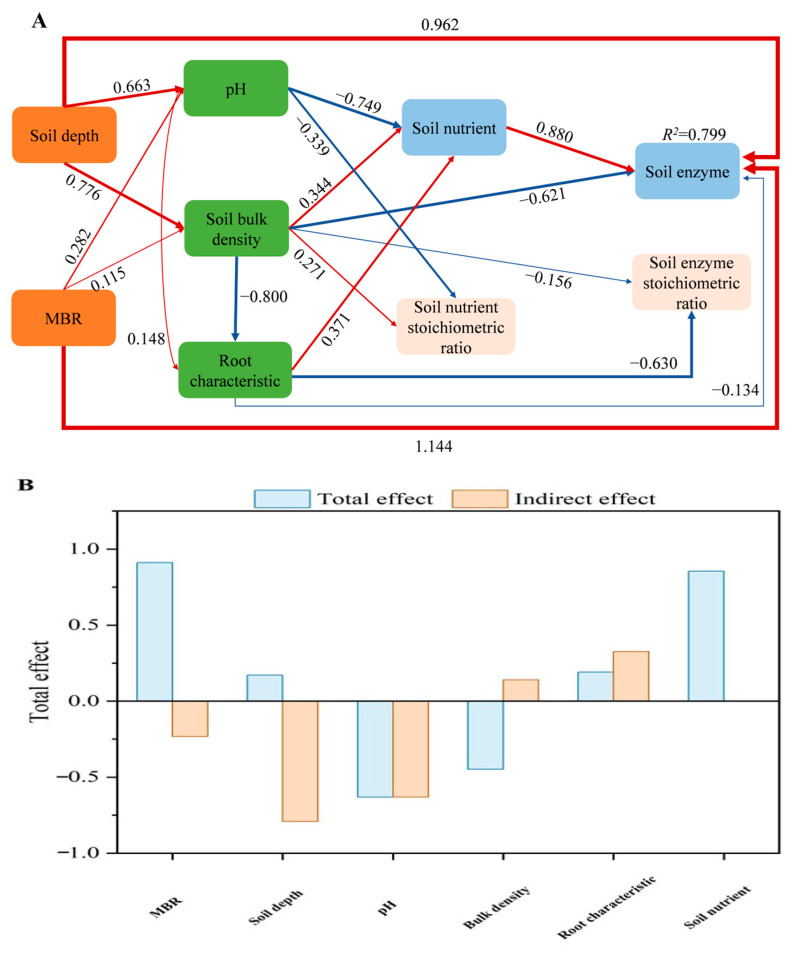
Structural equation model (SEM) for evaluating the direct and indirect effects on soil enzyme activities and the soil enzyme stoichiometric ratio (**A**) and their total effects (**B**) across soil depth and the MRB.

The Mantel test revealed a highly significant positive correlation between MRB and both C-acquiring enzymes and the E_C:N_ ratio. In contrast, TP exhibited exceptionally strong positive correlations with N-acquiring enzymes and the E_C:N_ ratio, indicating a significant relationship between phosphorus availability and nitrogen cycling processes in the soil system (Figure 4; *p* < 0.01). Moreover, N-acquiring enzymes were significantly positively correlated with the soil C:N and N:P ratios, indicating that coordinated nutrient dynamics are influenced by carbon and nitrogen cycling processes (*p* < 0.01). Specifically, the significant relationships between soil nutrients and enzymes in each MRB and soil depth demonstrated its key role in shaping enzymatic stoichiometry (Figure 5).

SEM analysis revealed the effects of root characteristics, soil nutrients, the soil nutrient stoichiometric ratio, soil depth, and MRB on the soil enzyme and soil enzyme stoichiometric ratios (Figure 5). The soil nutrient levels were significantly and positively correlated with the soil bulk density (path coefficient = 0.344) and root characteristics (path coefficient = 0.371) and showed an inverse relationship with the pH values (path coefficient = −0.749). Moreover, the soil nutrient content positively influenced the soil enzyme activity (path coefficient = 0.880) but negatively affected the soil pH (path coefficient = −0.749). Furthermore, soil enzyme activity was directly influenced by soil nutrients, soil depth (path coefficient = 0.962), and MRB (path coefficient = 1.144), indicating that these factors are key drivers of belowground biochemical processes. Soil enzyme activity was indirectly influenced by the soil bulk density (path coefficient = −0.621) and root characteristics (path coefficient = −0.134). In addition, the soil enzyme stoichiometric ratio was indirectly influenced by the soil bulk density (path coefficient = −0.156) and root characteristics (−0.630), indicating that physical soil properties and root system morphology play a role in regulating belowground biochemical processes.

### 3.5. Indicators of Microbial Resource Limitation

**Figure 6:**
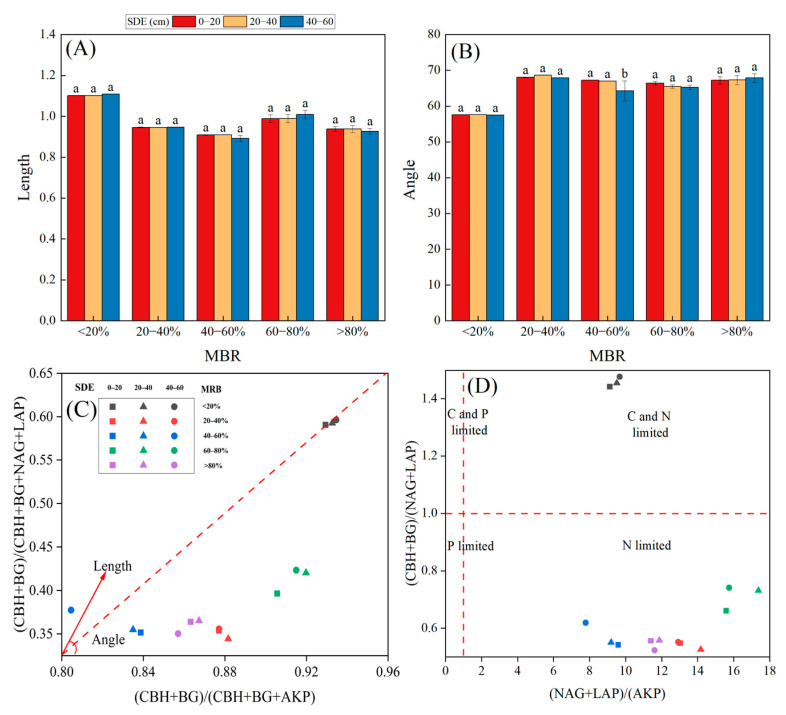
Changes in microbial resource limitation depended on the expansion ratios of bamboo at different soil depths within the broad-leaved forest. Soil enzyme stoichiometry of the variation in vector length and angle (**A**,**B**) and the relative proportions of C to N acquisition versus C to P acquisition (**C**,**D**). (**A**) represents the vector length indicating soil C limitation; (**B**) represents the vector angle indicating soil N/P limitation for microbes; (**C**) represents the enzyme activity ratios of carbon acquisition to (carbon + phosphorus) acquisition, expressed as (CBH + BG)/(CBH + BG + AKP), and the enzyme activity ratio of carbon acquisition to (carbon + nitrogen) acquisition, expressed as (CBH + BG)/(CBH + BG + NAG + LAP); (**D**) represents the enzyme activity ratio of carbon acquisition to phosphorus acquisition, expressed as (CBH + BG)/AKP, and the enzyme activity ratio of carbon acquisition to nitrogen acquisition, expressed as (CBH + BG)/(NAG + LAP). Different lowercase letters indicate significant differences among patches at the same soil depth (*p* < 0.05).

The vector length initially decreases and then increases as the MRB increases. Notably, the vector length reaches its peak in forests with the lowest MRB (<20%) (Figure 6), indicating that carbon limitation is most pronounced under the lowest MRB (< 20%), where it exceeds the values recorded in forests with MRB ranges of 20–40%, 40–60%, and 60–80% by factors of 1.35, 1.44, and 1.08, respectively. With the exception of the 20–40% MRB condition, where the vector length increases consistently with soil depth, the vector length initially increases and then decreases as the soil depth increases under all other MRB conditions. Specifically, the maximum vector length is observed at a soil depth of 20–40 cm, which corresponds to the greatest degree of C limitation. Additionally, the ratio of (CBH + BG) to (NAG + LAP) is less than 1 under all MRB conditions, except for the lowest MRB (<20%), where it exceeds 1. This suggests that both C and P limitations are present at the lowest MRB (<20%), whereas other MRB ranges exhibit only P limitation without C limitation. Furthermore, all the vector angles in the forest are greater than 45°, indicating that the soil in the bamboo forest is subject to phosphorus limitation. The vector angle increases gradually with increasing MRB, reaches the maximum value at 40–60% MRB, and then rapidly decreases to the minimum value at 60–80% MRB. Notably, all the vector angles at the 20–40 cm soil depth presented a minimum value. Overall, P limitation clearly reached its maximum at 60–80% MRB and at soil depths of 20–40 cm.

## 4. Discussion

### 4.1. Response of Root Characteristics and Soil Nutrients to MRB

The root system functions as the principal organ responsible for water and nutrient absorption in crops, and root morphological characteristics are critically important for the growth and development of bamboo forests. Our study revealed a progressive reduction in root morphology parameters with increasing soil depth, which is consistent with the findings of [18], who reported that the highest values of root weight density were consistently observed in shallow soil layers across all experimental treatments. This can be attributed to the progressive decrease in organic residues from fine roots in bamboo forests with increasing soil depth, which is accompanied by a concomitant decline in the diversity and abundance of soil microorganisms as well as in root physiological activity. Furthermore, this study revealed that bamboo forest root morphology was significantly less developed at both low (<20%) and high (≥80%) expansion ratios than at moderate expansion ratios (40–60%). This is primarily attributed to the relatively low expansion rates of bamboo forests, which lead to limited competition between bamboo root systems and broad-leaved forests for soil moisture and nutrients. Furthermore, isolated bamboo forest patches may lack the synergistic ecological advantages necessary for optimal growth and development, thereby reducing overall ecological functionality. In contrast, when the expansion ratio of bamboo forests is relatively high, excessive stand density intensifies competition for soil moisture and nutrients, resulting in reduced root length and root surface area due to resource limitations. High density may also inhibit photosynthesis through canopy shading, thereby decreasing the allocation of assimilates to the root system. Conversely, when the expansion ratio is moderate, bamboo forest density can promote the soil organic matter formation through the accumulation of litter and root exudates, thereby increasing soil water and nutrient retention [42,43].

Some studies have demonstrated that soil nutrients accumulate gradually during ecological succession from pure bamboo to mixed broad-leaved forests [14,38], with relatively high broad-leaved ratios increasing nutrient contents [32]. However, our study revealed that both the SOC and OM contents were relatively low under conditions of excessive or limited bamboo expansion, reaching maximum levels at 60–80% expansion. This trend is due mainly to the low expansion ratio of bamboo forests, which increases the leaf area index and reduces light penetration because of the greater abundance of broad-leaved trees and their wide crowns [44]. This decreases photosynthetic efficiency, slows the decomposition of litter, and reduces soil organic matter accumulation [45], which is consistent with the findings of [18]. When bamboo forest expansion exceeds 60% in karst regions, extensive rhizome growth in deep rock fissures blocks water access for broad-leaved tree roots, worsening water scarcity and inhibiting organic matter decomposition, as shown by [18]. In addition, our study revealed that surface soils contain high levels of nutrients (OM, SOC, TN, TP, and AP), as well as low pH, BD, and AN, which is consistent with the findings of [36]. This can primarily be attributed to the soil surface layer (0–20 cm) having a significantly greater influx of organic litter, as well as complexes of clay minerals and humus. Additionally, the intricate interweaving of bamboo rhizomes and fine roots from broad-leaved forests contributes to a reduction in BD, thereby establishing what may be termed a “shallow carbon pump” that enriches soil nutrients. With increasing soil depth, the litter input approaches zero, the root biomass and clay particle content decrease, while carbonate rock debris remains high, and the physical protection of organic matter weakens [26]. Additionally, the vertical migration of karst fracture water accelerated calcite dissolution and HCO_3_^−^ release, leading to a gradual increase in pH. Moreover, owing to the hypoxic conditions and elevated pH levels in the deep soil layers, the processes of denitrification and ammonia volatilization significantly increased, whereas the mineralization rate of organic nitrogen significantly decreased [46]. In general, surface soils host a rich nutrient, low pH active carbon pool, whereas deeper layers constitute a high pH, depleted nutrient inert zone dominated by geochemical processes.

### 4.2. Response of Soil Enzyme Activities and Microbial Resource Limitations in the MRB

Soil enzyme activity serves as a critical indicator of soil ecological function, directly mediating nutrient cycling and organic matter decomposition [27,47]. This study revealed that the surface soil layer (0–20 cm depth) presented the highest levels of all soil enzyme activities at 60–80% MRB, which is similar to the findings of [48], who reported a decrease in soil enzyme activity with increasing soil depth. This trend could be attributed to favorable water and heat conditions, as well as good ventilation at the soil surface. These conditions provide the necessary nutrients for microbial growth, leading to vigorous microbial activity. The active metabolism of microorganisms results in high enzyme activity in the surface soil. However, when the MRB was less than 60%, the C- and P-acquiring enzyme activities were highest at a soil depth of 40–60 cm and lowest at 20–40 cm. This observation differs from the findings of [49], who found that surface soils are more favorable for enzyme activity. This discrepancy may stem from the accumulation of litter with less than 60% MRB in the surface layers, increasing fertility there. As surface soil enzyme activity stabilizes, deeper layers show increasing activity and may eventually exceed surface levels because of leaching and deposition from upper layers. Notably, we observed that C-acquiring enzyme activities (CBHs + BGs) were highest at <20% MRB, whereas N- and P-acquiring enzyme activities were highest at 20–40% MRB. Moreover, the soil enzyme activities gradually decreased with increasing MRB. These findings indicate that the soil carbon, nitrogen and phosphorus cycles are faster when broad-leaved forests are more abundant [33,50]. This phenomenon is due mainly to broad-leaved forests producing abundant organic litter, which supports rich tree fruit production and enhances microbial and biological activity throughout the forest ecosystem [2]. Simultaneously, a considerable portion of broad-leaved forests contain root secretions from diverse tree species, which provide copious energy for the metabolic processes of soil microorganisms while also modulating the activity of soil enzymes, thereby stimulating a more substantial release of these enzymes. Moreover, our study revealed that AKP activity was lower at 60–80% *MRB* than at the other *MRB* concentrations. This phenomenon may be attributed to the extraordinarily high density of bamboo forests and the substantial vegetation biomass within the forest ecosystem, which suppresses soil acidic phosphatase activity in the forest. Specifically, the expansion ratios of bamboo in mixed forests reduce canopy gaps, thereby limiting light penetration and resulting in relatively cool soil temperatures, which collectively inhibits the metabolic activities of soil microorganisms [3]. Moreover, the high calcium and alkaline characteristics of karst soils promote the association of phosphate with calcium ions, leading to the precipitation of insoluble calcium phosphate [13]. This condition, combined with the limited available phosphorus, inhibits microbial activity and reduces the secretion of enzymes associated with phosphate mineralization, such as phosphatases. Consequently, these interconnected factors significantly decrease soil AKP activity.

On the basis of resource allocation theory, soil microorganisms can mitigate soil nutrient limitations by modulating enzyme secretion [4,16]. Therefore, examining the response patterns of soil enzyme stoichiometric ratios to different *MBA* values and soil depths can provide valuable insights into the adaptive strategies employed by soil microorganisms to address nutrient limitations in karst mixed bamboo and broad-leaved forests. Furthermore, our study revealed that when the *MBA* was <20%, the E_C:P_ and E_C:N_ ratios were significantly greater, whereas the E_N:P_ ratio was relatively lower. Conversely, when the expansion ratio of broad-leaved forests is significantly greater than that of bamboo forests, microorganisms secrete a substantial amount of C- and P-related soil enzymes. This suggests that soil microbial growth may be limited by the availability of C and P, thereby emphasizing the efficient resource acquisition mechanisms utilized by microorganisms for these elements. However, as the expansion ratios of bamboo forests become harmoniously integrated into broad-leaved forests, the diversity of plant litter diversifies, establishing an optimal equilibrium of light and water resources in karst terrain. Beneath the surface, a complex underground network forms in which shallow roots are intricately intertwined with deep roots. Concurrently, plant roots release a more diverse array of secretions, fostering a vibrant and dynamic ecosystem [4,48]. Therefore, when bamboo forests continue to expand within broad-leaved forests, the ratios of soil E_C:P_, E_C:N_, and E_N:P_ gradually decrease. Moreover, our study revealed that the E_C:N_ ratio (0.92) was significantly lower than the global average of E_C:N_ (1.41), whereas both the E_C:P_ ratio (2.31) and E_N:P_ ratio (2.51) were significantly higher than their respective global averages (0.62 and 0.44 for E_C:P_ and E_N:P_, respectively) [51]. This is because low temperatures suppress the decomposition and transformation efficiency of soil microorganisms with respect to litter in high-altitude regions (>1500 m), resulting in a slow turnover rate of soil organic carbon (SOC). However, the high productivity of bamboo forests produces a large quantity of litter with a low C:N ratio; during decomposition, nitrogen is rapidly retained in the soil, whereas carbon is preferentially released as CO_2_, thereby further reducing the C:N ratio. Furthermore, karst ecosystems are recognized as typical phosphorus-limited systems globally. The rapid expansion of *Fargesia denudata* intensifies the demand for soil phosphorus, leading to a further decline in available P. Concurrently, the substantial nitrogen inputs and relatively greater accumulation of carbon associated with *Fargesia denudata* significantly increase the amounts of carbon and nitrogen per unit of phosphorus in the soil [10,21]. Moreover, all the vector angles of the soil enzymes in our study exceeded 45°, further confirming the low phosphorus availability in mixed bamboo and broad-leaved forests across the karst region. Moreover, we revealed that, in comparison with those of the other *MBAs*, the vector lengths of the soil enzymes for the lowest MRB (<20%) were relatively longer, whereas the vector angles of the soil enzymes were relatively smaller. This further indicates that when broad-leaved forests are in absolute dominance, the soil is subject to more pronounced limitations in C and P availability than soils associated with higher expansion ratios of bamboo forests. This phenomenon can be attributed to the dominance of broad-leaved forests in karst regions, where forests are generally relatively old, and the weathering-induced release of nutrients from the parent material progressively diminishes over time. Additionally, broad-leaved forests typically exhibit substantial biomass but lack an efficient mycorrhizal symbiosis system [2]. As a result, despite the high phosphorus demand in broad-leaved forests, their phosphorus absorption efficiency remains relatively low, thereby exacerbating phosphorus limitation [3,40]. Through the secretion of phosphatase, these systems can activate insoluble phosphorus, thereby alleviating nutrient limitations in forest ecosystems. It can be concluded that when the MBA is 40–60%, the soil nutrient limitation will achieve optimal alleviation.

## 5. Conclusions

We conducted an in-depth study to investigate soil enzyme activities and their stoichiometric ratios in relation to soil depth and bamboo expansion ratios within a monsoon broad-leaved forest (MRB) in a karst region. Our results indicated that the FRW, RLD, RSAD, and RVD decreased with increasing soil depth. Moreover, the root morphology was significantly less developed at both low (<20%) and high (≥80%) expansion ratios than at moderate expansion ratios (40–60%). SOC and OM increased with MRB from 0% to 60%, reaching their minimum values at 60–80% MRB. Moreover, the root morphology was significantly less developed at both low (<20%) and high (≥80%) expansion ratios than at moderate expansion ratios (40–60%). SOC and OM increased with MRB from 0% to 60%, reaching their minimum values at 60–80% MRB. Moreover, when the MRB was below 60%, C- and P-acquiring enzyme activities were highest at the 40–60 cm soil depth and lowest at the 20–40 cm soil depth. Our study revealed that the E_C:N_ ratio (0.92) is significantly lower than the global average (1.41), whereas both the E_C:P_ (2.31) and E_N:P_ (2.51) ratios are markedly higher than their respective global averages (0.62 and 0.44). This indicates a relative scarcity of soil phosphorus and nitrogen, prompting microorganisms to produce greater amounts of N- and P-degrading enzymes to satisfy metabolic requirements. Furthermore, Mantel tests and structural equation modeling (SEM) analyses demonstrated that soil enzyme activity is directly influenced by soil nutrients, depth, and MRB and indirectly influenced by bulk density and root traits. Therefore, our findings highlight the critical roles of MRB and soil depth in regulating nutrient cycling in karst ecosystems, where bamboo forest expansion primarily influences soil enzyme activities and microbial resource limitation through changes in root traits, soil bulk density, and nutrient availability.

## Figures and Tables

**Table 1:** Root characteristics of bamboo as a function of the expansion ratio within the broad-leaved forest.

MRB (%)	Soil Depth (cm)	Fresh Root Weight (g)	Dry Root Weight (g)	Root Biomass Density (g·m^−3^)	Root Length Density (mm·m^−3^)	Root Surface Area Density (cm^2^·m^−3^)	Root Volume Density (cm^3^·m^−3^)
<20%	0–20	5.04 ± 3.48 a	1.57 ± 0.47 a	2 ± 0.59 a	0.88 ± 0.75 a	0.16 ± 0.14 a	2.44 ± 2.13 a
	20–40	3.8 ± 5.02 a	1.49 ± 2.13 a	1.9 ± 2.71 a	0.31 ± 0.24 a	0.06 ± 0.04 a	0.95 ± 0.51 b
	40–60	0.86 ± 0.43 a	0.21 ± 0.12 a	0.26 ± 0.16 a	0.15 ± 0.07 a	0.03 ± 0.01 a	0.61 ± 0.26 b
20~40%	0–20	26.99 ± 2.51 a	9.03 ± 7.06 a	11.51 ± 8.99 a	1.89 ± 1.4 a	0.42 ± 0.27 a	7.4 ± 4.1 a
	20–40	9.91 ± 7.92 a	3.13 ± 2.73 a	3.98 ± 3.48 a	1.26 ± 1.27 a	0.3 ± 0.25 a	5.87 ± 3.77 a
	40–60	1.11 ± 0.72 a	0.28 ± 0.23 a	0.35 ± 0.29 a	0.24 ± 0.28 a	0.05 ± 0.05 a	0.78 ± 0.72 b
40~60%	0–20	11.6 ± 8.66 a	2.95 ± 2.07 a	3.75 ± 2.64 a	1.62 ± 0.85 a	0.42 ± 0.24 a	8.76 ± 5.44 b
	20–40	22.99 ± 3.79 a	10.15 ± 18.12 a	12.94 ± 23.09 a	0.71 ± 0.34 b	0.27 ± 0.22 ab	10.19 ± 13.35 a
	40–60	0.89 ± 0.14 a	0.39 ± 0.24 a	0.5 ± 0.3 a	0.27 ± 0.09 b	0.06 ± 0.03 b	1.22 ± 0.82 c
60~80%	0–20	15.42 ± 3.56 a	3.15 ± 2.61 a	4.02 ± 3.33 a	1.63 ± 1.22 a	0.38 ± 0.3 a	7.17 ± 5.98 b
	20–40	15.24 ± 12.10 a	4.92 ± 4.04 a	6.27 ± 5.14 a	0.42 ± 0.14 a	0.16 ± 0.04 a	5.4 ± 2.76 b
	40–60	4.66 ± 6.03 a	1.38 ± 1.8 a	1.75 ± 2.29 a	0.23 ± 0.06 a	0.1 ± 0.06 a	12.25 ± 13.13 a
>80%	0–20	11.96 ± 3.96 a	3.34 ± 1.37 a	4.25 ± 1.74 a	1.51 ± 0.8 a	0.34 ± 0.18 a	6.21 ± 3.4 a
	20–40	1.37 ± 0.61 b	0.53 ± 0.24 b	0.68 ± 0.31 b	0.33 ± 0.17 b	0.09 ± 0.04 b	1.87 ± 0.97 b
	40–60	0.49 ± 0.06 b	0.11 ± 0.06 b	0.14 ± 0.002 b	0.12 ± 0.02 b	0.03 ± 0.01 b	0.5 ± 0.09 b

Note: Different lowercase letters (follow by data) indicate significantly differ between different landscapes at *p* < 0.05 level.

## Data Availability

The data used in this study are confidential; however, all data can be made available upon reasonable request by contacting the corresponding author.
